# Advances in the Study of the Antiatherogenic Function and Novel Therapies for HDL

**DOI:** 10.3390/ijms160817245

**Published:** 2015-07-28

**Authors:** Peiqiu Cao, Haitao Pan, Tiancun Xiao, Ting Zhou, Jiao Guo, Zhengquan Su

**Affiliations:** 1 Key Research Center of Liver Regulation for Hyperlipemia SATCM/Class III, Laboratory of Metabolism SATCM, Guangdong TCM Key Laboratory for Metabolic Diseases, Guangdong Pharmaceutical University, Guangzhou 510006, China; E-Mails: cpq_520@126.com (P.C.); pangel7835001@163.com (H.P.); 2 Inorganic Chemistry Laboratory, University of Oxford, South Parks Road, Oxford OX1 3QR, UK; E-Mail: xiao.tiancun@chem.ox.ac.uk; 3 Guangzhou Boxabio Ltd., D-106 Guangzhou International Business Incubator, Guangzhou 510530, China; E-Mail: ting4677@126.com

**Keywords:** HDL, biomarker, HDL function, reverse cholesterol transport, HDL therapies

## Abstract

The hypothesis that raising high-density lipoprotein cholesterol (HDL-C) levels could improve the risk for cardiovascular disease (CVD) is facing challenges. There is multitudinous clear clinical evidence that the latest failures of HDL-C-raising drugs show no clear association with risks for CVD. At the genetic level, recent research indicates that steady-state HDL-C concentrations may provide limited information regarding the potential antiatherogenic functions of HDL. It is evident that the newer strategies may replace therapeutic approaches to simply raise plasma HDL-C levels. There is an urgent need to identify an efficient biomarker that accurately predicts the increased risk of atherosclerosis (AS) in patients and that may be used for exploring newer therapeutic targets. Studies from recent decades show that the composition, structure and function of circulating HDL are closely associated with high cardiovascular risk. A vast amount of data demonstrates that the most important mechanism through which HDL antagonizes AS involves the reverse cholesterol transport (RCT) process. Clinical trials of drugs that specifically target HDL have so far proven disappointing, so it is necessary to carry out review on the HDL therapeutics.

## 1. Introduction

Hyperlipidemia, a risk factor for atherosclerosis (AS), is a serious consequence for people who have experienced coronary heart disease, stroke, and artery stenosis disease. AS, which is the leading cause of cardiovascular disease (CVD), is responsible for 50% of all mortality in many developed countries [1]. A persistent increase in circulating low-density lipoprotein cholesterol (LDL-C) levels in the body is one of the most important causes for the initiation and progression of AS [2]. It has been shown in epidemiological studies and clinical trials that LDL-C levels are directly related to the rate at which CVD events occur [2,3]. There are abundant antilipemic agents (Table 1) on the market, but the meta-analysis of intervention trials has shown that a per mmol/L decrease in LDL-C is associated with an approximate 22% reduction of CVD events and a 10% reduction of all-cause mortality [3]. There is a wealth of evidence showing that statins that play a beneficial role in lowering LDL-C levels and are efficient in preventing first cardiovascular events. However, a large residual disease burden remains, even in patients treated with a high dose of statins and other CVD risk-modifying interventions [1]. Furthermore, treatment with statins may lead to a significant increase of muscle toxicity and liver transaminase, and may not be suitable for all CVDs. Investigators are eagerly searching for novel therapeutic targets. Because high-density lipoprotein cholesterol (HDL-C) levels, a predictor of major cardiovascular events in patients, are inversely associated with risk for CVD [2,4], strategies for increasing HDL levels have been explored as a new approach for combatting CVD, which may overcome the significant residual cardiovascular risk remaining after treatment with statins [4]. However, a recent genetic analysis failed to show a causal association between genetically raised plasma HDL cholesterol levels and risk for myocardial infarction [5]. In addition, nicotinic acid and fibrates currently are used to increase HDL-C levels, both of which have some weaknesses (e.g., uricosuria, increased glucose tolerance and flushing for nicotinic acid and problematic pharmacokinetic interactions for fibrates) that impose restrictions on their use [6]. Moreover, raising HDL-cholesterol by the cholesteryl-ester transfer protein (CETP) inhibitor did not play its expected role in protection from CVD [7,8]. The goals of previous HDL therapy are currently being reassessed due to numerous difficulties validating the hypothesis. Therefore, HDL structure, composition and function are the focus of ongoing research efforts, as they might provide more valuable information than steady-state HDL-cholesterol levels [9]. From the study of the structure and composition of HDL, novel strategies for the treatment of hyperlipemia-induced AS are being developed. Apolipoprotein A-I (apoA-I) is the major structural protein component of HDL particles [10], and it has been shown to play a pivotal role in reverse cholesterol transport (RCT) [11]. ApoA-I has also been shown to exert direct anti-inflammatory effects [12]. Stimulating increased synthesis of endogenous apoA-I may be a promising approach. Regarding the function of HDL, the atheroprotective activities of HDL particles are attributed to their central role in anti-inflammatory, antithrombotic, and antioxidant processes and their ability to improve endothelial function [13,14]. In addition, RCT is currently understood as the physiological process by which cholesterol in peripheral tissues is transported by HDL to the liver for excretion in the bile and feces [15]. This process is complex and beneficial, and it has been known as a widely accepted mechanism for the protective effect of HDL.

**Table 1:** Drugs of anti-hyperlipidemia in the current market. In recent years many cholesterol-lowering drugs are commonly used on the market, which mainly includes stains, fibrates, nicotinic acids, cholesterol absorption inhibitor and polyene unsaturated fatty acids.

Classification	Drug	Mechanism	TC	TG	VLDL	LDL	HDL	Advantage	Disadvantage
Stains	Lovastatin	As inhibition of HMG CoA reductase, reduce cholesterol synthesis		**↓**	**↓**	**↓**	**↑**	The advantage of these drugs is a low incidence of adverse reaction, and can be suitable for a variety of hypercholesterolemia except hypertriglyceridemia , is a lipid-lowering drug with rapid development in recent years	Gastrointestinal symptoms and rash, and the residual risk
	Atorvastatin					**↓**			
Fibrates	Gemfibrozil	The drug can increase Lp(a) Lipase activity to remove VLDL, TG;		**↓**	**↓**	**↓**	**↑**	These drugs do not cause the increase of diabetic insulin resistance or affect the control of blood sugar, therefore this kind of drugs is the first choice for treating the diabetic patients with hyperlipidemia	Gastrointestinal reactions, allergic reaction, due the drugs increase the concentration of cholesterol in bile, it may cause gallstones, occasional eyesight obstacle and hematological abnormalities
	Fenofibrate	Thus reducing VLDL and TG, TC and LDL can also be reduced		**↓**					
Nicotinic Acids	Niacin; Inositol Aluminum	The drug can prevent fat decomposition, prevent free fatty acid formation, inhibit synthesis of TG and secretion of VLDL in liver		**↓**	**↓**	**↓**	**↑**	Cheap, and it is the only lipid-lowering drug can also reduce risk and mortality of cardiovascular disease	It is not suitable for diabetes patients, overdose adverse reactions (toxic to the liver, high blood sugar) has a high incidence common adverse reactions are skin flushing, itching, rash
	Aluminum Nicotinate					**↓**			
Cholesterol Absorption Inhibitor	Ezetimibe	The drug can combine with bile acid to block the bile acid absorption;	**↓**	**↓**		**↓**	**↑**	This kind of medicine is recognized as TC lowering drugs, when treats together with statins, the risk of accidental heart disease related to decrease the occurrence of 50% or more	The common adverse reactions are mild nausea and abdominal distension, constipation, therefore, it is not suitable for intestinal diseases and intractable constipation patients
		Prompte the translation of the cholesterol into bile in the gallbladder, then bile binding to drug is eliminated from the body	**↓**						
Polyene Unsaturated Fatty Acids	Duoxikang	This drug can combine with total cholesterol to be ester; Then promotes the degradation of bile acid excreted along with the bile, decreases plasma total cholesterol concentration	**↓**	**↓**		**↓**		These drugs in combination with statins can reduce the level of TG, and play an effective role in the prevention and treatment of coronary heart disease	This kind of medicaments is easy to be oxidized to atherogenic substance, has inhibitory effect on platelet aggregation, so it needs to be used with caution
	Ecosapeatanolic acid								
				**↓**					

This paper is aimed at looking for a better HDL biomarker to predict AS precisely and explaining how HDL functions in detail, then providing an overview of novel therapies that target HDL to enhance HDL’s ability to reduce residual cardiovascular risk in the population. Finally, we carry out an assessment of a natural drug that has been linked to HDL, which represents a unique field in the treatment of AS that warrants exploration.

## 2. HDL and AS

AS can be considered to be a form of chronic inflammation, beginning with increased endothelial permeability of monocytes under the influence of adhesion molecules [16]. It promotes endocytosis of oxidized LDL into the arterial wall and intima, which can be devoured by intimal macrophages to become foam cells. This is a key process in the development of atherosclerotic plaque. Along with an increase in macrophages and foam cells, the plaque becomes unstable. This progression can ultimately lead to the development of complex lesions, or plaques, that protrude into the arterial lumen. Plaque rupture and thrombosis result in the acute clinical complications of myocardial infarction and stroke [17,18]. Cardiovascular diseases are the leading cause of death and illness in developed countries, with AS being the most important contributor [13]. In recent years, there has been growing interest in finding a cardiovascular biomarker that provides prognostic and predictive information to act as a tool to influence treatment strategies [19]. The use of HDL-related indexes as biomarkers is undergoing predictive tests, and they represent the greatest promise of this technology and the shortest and most effective path to furthering our understanding.

### 2.1. HDL as the Biomarker for AS

#### 2.1.1. HDL-C

Because trends relating high plasma levels of HDL-C and decreased incidence of CVD endpoints were observed in prospective epidemiological studies conducted in several countries many years ago, HDL-C levels have served as a significant predictor for relieving AS in the clinic. There have been several attempts to increase HDL-C levels using pharmacological intervention [3,17]. HDL-C levels have been reported to increase upon chronic administration of fibrates as agonists of the peroxisome proliferator activated receptor α (PPARα) in animals and in humans. Niacin is the first antidyslipidemic agent identified that has been available for patients to raise HDL-C. However, recent data from a Global Health outcomes (AIM-HIGH) trial with niacin did not show any significant improvement in the cardiovascular risk over statins, which are commonly used drugs for lipid management [20,21]. Recently a few attempts have been made to inhibit CETP to raise the levels of HDL-C. Torcetrapib, a cholesterol ester transferase inhibitor, reliably increased HDL (without countervailing effects on LDL) but ultimately increased cardiovascular mortality. Dalcetrapib, a new CETP inhibitor, also has unintended effects, and the outcomes are the same as those for torcetrapib [7,8]. As a result, CETP inhibitors continue to face safety hurdles, and no significant clinical benefits have resulted from these pharmacological regimens. A recent report suggested that the sole increase of HDL-C in humans would not necessarily improve the rate of cardiovascular events (all-cause mortality, coronary heart disease mortality, non-fatal myocardial infarction, and stroke) [22,23,24,25]. In justification for the use of statins in prevention: an Intervention Trial Evaluating Rosuvastatin (JUPITER), on-treatment HDL-C was not predictive of residual risk among statin-treated individuals, whereas HDL-C was predictive among those taking placebo [26]. Similarly, on-treatment apoA-I and triglycerides were not predictive of residual risk [27]. For these reasons, the use of the HDL-C biomarker as a surrogate end point remains a difficult and distant goal.

#### 2.1.2. HDL Particle Size

Dyslipidemia may influence enzymes and transfer proteins needed for lipoprotein particle remodeling. HDL particle size may differ in the number of molecules of apo and free cholesterol, esterified cholesterol, and phospholipids content on the lipoprotein surface [28,29] due to the changes in activity of Lecithin-cholesterol acyltransferase (LCAT), CETP, phospholipid transfer protein (PLTP) plasma transfer proteins, and enzymes (e.g., lipoprotein lipase (LPL), hepatic lipase (HL)) in the process. Calculated indices and the evaluation of lipoprotein particle size have been widely used to predict cardiovascular risk. An evaluation of the association between HDL particle size and risk of incident coronary artery disease (CHD) in apparently healthy volunteers indicated that decreased HDL particle size is associated with an adverse cardiometabolic risk profile [30]. Small HDL particle size was also associated with an increased CHD risk, which resulted from the different lipid transfer ability [31]. Another study found that the high TG-low HDL cholesterol dyslipidemia, which is found in viscerally obese subjects and characterized by hyperinsulinemia, was strongly correlated with reduced HDL particle size [32]. However, the latest finding is not consistent with previous conclusions. The HDL particle size (nm) values were not different between the dyslipidemia, normolipidemic and dyslipdemic groups without treatment with lipid-lowering drugs [33]. In addition, several studies using GGE-measured HDL size have reported that patients with CHD tend to have smaller HDL particles and that large HDL particles may protect against the development of AS. There are also other studies that show that only small HDL particles are atheroprotective, this is supported by the analysis of HDL subfraction data and carotid artery disease (CAAD) [34]. It is found that among HDL-C, HDL_2_, and HDL_3_, HDL_3_ best predicts CAAD risk, and the remaining phenotypes do not add significant predictive power [34]. Although public opinions about HDL size are divergent, the propagation rate and maximal diene formation during total HDL oxidation correlated significantly with HDL mean particle size [30,32]. It is clear that proper HDL particle size may have advantages in the reduction of CVD events. In the future, we believe that HDL particle size will play an essential role in predicting the risk of AS.

#### 2.1.3. HDL Particles Concentration (HDL-P)

Given the extreme heterogeneity of HDL, measuring the content of HDL particles will, at best, only partially reflect the potential role of HDL in cardiovascular risk assessment and therapeutic drug development. In this regard, the HDL-P may be a better marker of residual vascular risk after potent statin therapy in the JUPITER trial than chemically measured HDL-C or apoA-I [35]. HDL-P may be a promising metric of HDL that is more independent of other metabolic and lipoprotein risk factors than HDL cholesterol or HDL size [36]. In the European Prospective Investigation into Cancer and Nutrition (EPIC)-Norfolk study, HDL-P was inversely associated with CVD, consistent with the above-mentioned observations [37]. However, in the Women’s Health Study, HDL-P was not associated with incident CVD events among healthy low-risk women in contrast with inverse associations seen for HDL size and HDL-C [38]. Moreover, an emerging outcome from a determinant of residual risk among statin-treated individuals suggests that overall HDL-P is not as important as the sub-type of HDL-P, which contains the lysosphingolipid sphingosine-1-phosphate (S1P) [36]. This conclusion is supported by the evidence that it is the S1P content of HDL, as opposed to the HDL particle itself, which is responsible for the beneficial antiatherothrombotic, anti-inflammatory, antioxidant, antiglycation, and profibrinolytic activities of these lipoproteins [36]. At present, two techniques, nuclear magnetic resonance (NMR) and ion mobility analysis (IMA) have been described for quantifying HDL-P in human plasma. However, the final HDL-P yield determined using these two methods differ by up to >5-fold. With the progress of science and technology, the accurate and reasonable use of ^1^H NMR [39] and application of termed calibrated ion mobility analysis (calibrated IMA) [40] will greatly determine the feasibility of HDL-P as biomarker.

#### 2.1.4. Other Biomarkers

The HDL-associated Apolipoprotein M (apoM) plays a role in the anti-atherogenic function in a variety of atherosclerotic models, making it interesting to investigate whether apoM is a predictor of AS [41,42,43,44]. With the discovery of apoM as an important carrier of S1P in HDL particles, it forms a new basis for investigations of apoM biology. An improved understanding of the role of the apoM/S1P axis in relation to AS may unravel new avenues for treatment or use of biomarkers for disease or risk evaluation [45].

The expression of serum amyloid A (SAA) protein was greater in the patients with AS, and it can be explained by high serum levels of SAA-predicted AS [45,46,47,48,49]. By means of two-dimensional electrophoresis (2-DE) coupled with mass spectrometry (MS) analysis on plasma-purified VLDL, LDL and HDL fractions from patients undergoing carotid endarterectomy, increased levels of acute-phase SAA (AP SAA) in all lipoprotein fractions helped to identify AP SAA as a potential marker of advanced carotid AS [50]. Studies in both mice and humans suggest that proinflammatory HDL may be a novel biomarker for increased risk of AS in patients with systemic lupus erythematosus (SLE) and rheumatoid arthritis (RA) [51]. Moreover, proinflammatory HDL-associated hemoglobin (Hb) was also found to be differentially associated with HDL from coronary heart disease patients compared with healthy controls [52,53]. Hb contributes to the proinflammatory nature of HDL in mouse and human models of AS and may serve as a novel biomarker for AS [52,53,54].

In addition, HDL has a wide range of functions, some of which are independent of its cholesterol content. Tests for HDL function as a biomarker may be useful for diagnosing the risk for patients with CHD. As a result, we elaborate the functions of HDL in the next section with the purpose of identifying the best biomarker of AS.

### 2.2. HDL in Anti-Atherosclerosis

**Figure 1:**
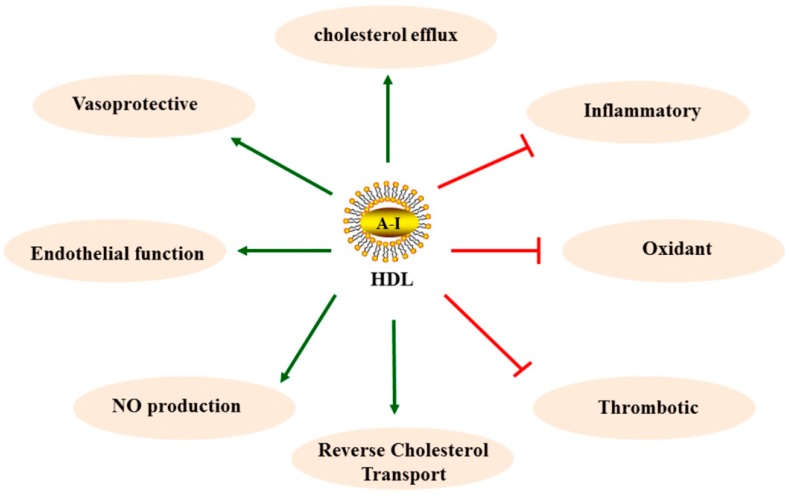
HDL antiatherosclerotic functions. 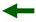 (promotion), 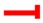 (inhibition).

HDLs have several well-documented functions with the potential to protect against AS. These functions include an ability to promote the efflux of cholesterol from macrophages in the artery wall, inhibit the oxidative modification of LDLs, inhibit vascular inflammation, inhibit thrombosis, promote endothelial repairing, promote angiogenesis, enhance endothelial function, improve diabetic control, and inhibit hematopoietic stem cell proliferation. Some of these functions have been mechanistically linked to the well-known ability of HDLs to induce the activation of cellular cholesterol efflux pathways, whereas many other functions of HDLs are independent of the effects of HDLs on cellular cholesterol homeostasis. Below, we will discuss the most relevant functions of HDLs for AS more specifically (Figure 1).

#### 2.2.1. Reverse Cholesterol Transport

**Figure 2:**
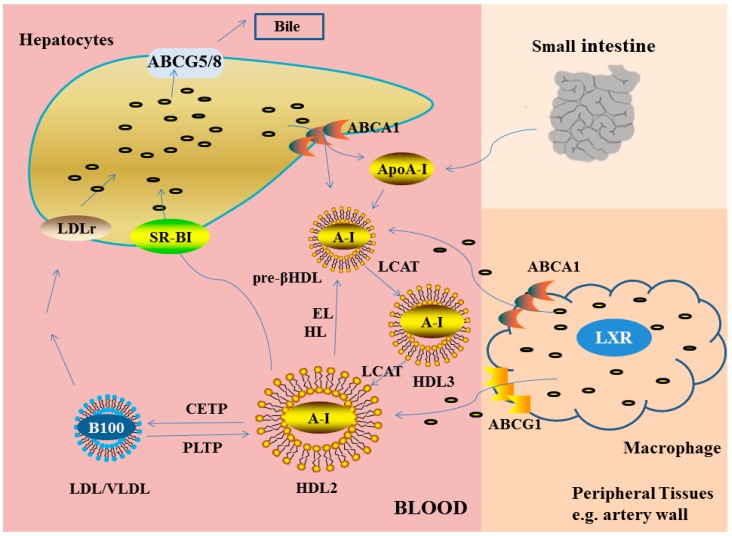
HDL in reverse cholesterol transport pathway. Lipid-poor apoA-I also promotes the efflux of free cholesterol from macrophages via ABCA1. LCAT esterifies free cholesterol to cholesteryl esters to form mature HDL, which promotes cholesterol efflux from macrophages via the ABCG1 transporter, as well as from other peripheral tissues by processes not fully defined. In macrophages, both ABCA1 and ABCG1 are regulated by Liver X receptors (LXR). Mature HDL can transfer its cholesterol to the liver directly via scavenger receptor class B type I (SR-BI) or indirectly via CETP-mediated transfer to ApoB-containing lipoproteins, with subsequent uptake by the liver via the LDL-r. Hepatic cholesterol can be excreted directly into the bile as cholesterol or after conversion to bile acids and, unless reabsorbed by the intestine, is ultimately excreted in the feces. HL, EL, and PLTP, play an indispensable role in remodeling HDL, thus, the RCT pathway is dependent on interaction with them [9].

In many studies, we found that the ability of HDL to promote cholesterol efflux from macrophages was strongly and inversely associated with both subclinical AS and obstructive coronary artery disease [55,56]. The efflux of cholesterol from a variety of cell types, including macrophages, to HDLs in the extracellular space is mediated by two distinct processes. One is the efflux of cholesterol induced by a specific cellular transporter [57,58,59,60,61,62], and the other is passive aqueous diffusion of cholesterol from cell membranes to HDLs [63,64]. Then, excess cholesterol from peripheral tissues will be transported back to the liver for excretion in the bile and ultimately the feces by HDL via a process called RCT. We herein would like to describe the importance of RCT in detail, and therefore, the multiplex RCT pathway has been described in the following four parts (Figure 2) [65].

##### Part One: The Formation of Nascent HDL

Lipid-free or lipid-poor apoA-I secreted in the liver can mediate cellular efflux of both cholesterol and phospholipids from macrophages via the ATP-binding cassette transporter A1 (ABCA1) and gather them on the surface of pre-β HDL, resulting in the rapid lipidation of apoA-I to generate mature α HDL, which is called nascent HDL particles [66,67]. Once loss of two ABCA1 genes occurs in the body, such as in the case of Tangier disease, the result is abnormally low levels of serum HDL-C, an acceleration of the accumulation of cholesterol in peripheral tissues, and the formation of premature AS [68]. The mature HDL particles can then serve as acceptors of cholesterol provided by ATP-binding cassette transporter G1 (ABCG1) [69] or SR-BI [70]. ABCG1 is another member of the ATP-binding cassette family that plays a critical role in the efflux of cellular phospholipid (PL) and free cholesterol (FC) to mature HDL, but not pre-β HDL. By intraperitoneal injection of mice with [^3^H]-cholesterol-labeled J774 macrophages with either increased or reduced ABCG1 expression and primary macrophages lacking ABCG1 expression, followed by measurement of the macrophage-derived [^3^H]-cholesterol levels in plasma and feces [71], it was shown that macrophages lacking ABCG1 cause damage and secretion of FC and excessive cholesterol accumulation in macrophages. Therefore, ABCG1 mediates cholesterol efflux [72]. In an investigation of extracellular cholesterol microdomains that form during the enrichment of macrophages with cholesterol, extracellular cholesterol microdomains did not develop when ABCG1-deficient mouse bone marrow-derived macrophages were enriched with cholesterol [69]. Many studies have demonstrated that ABCA1 and ABCG1 play a role in many aspects of cholesterol efflux from macrophages [69,71,72,73].

Liver X receptors (LXR), the members of the steroid nuclear receptor superfamily, are oxysterol-activated transcription factors that, after heterodimerization with the 9-*cis*-retinoic acid receptor (RXR), bind to specific LXR response elements (LXREs), thus regulating the expression of target genes involved in intra- and extracellular lipid metabolism [74]. In part by modulating cholesterol efflux from macrophages to apoA-I and HDL, LXRs induce the direct target genes ABCA1 and ABCG1/ABCG4 to promote reverse cholesterol transport. The oxidation of steroids from FC can activate LXR and regulate gene expression of ABCA1 and ABCG1 to strengthen the peripheral tissue cholesterol secretion. Meanwhile, the LXRs are also readily oxidized by PPARα. PPARαcontrols lipid and glucose metabolism in several tissues and cell types including liver, heart, kidneys, adipose tissue and macrophages. PPARα-activation suppresses chylomicron and increases HDL production by enterocytes [75,76]. In addition, its agonists promote secretion of macrophage cholesterol via stimulating expression of ABCA1 and LXR to increase reverse cholesterol transport [77].

##### Part Two: The Process of Cholesterol Esterified by LCAT

LCAT, a lipoprotein-associated enzyme, is a key player in the RCT pathway. LCAT has two different catalytic activities that account for its ability to esterify cholesterol. One is phospholipase A2 activity, and the other is its transesterification activity. It requires apoA-I and, to a lesser degree, other apolipoproteins, which most likely activate LCAT by modifying the presentation of its substrates, namely, phospholipids and cholesterol, on the surface of lipoproteins [78]. After FC efflux to pre-β HDL (the nascent, discoidal-shaped HDL), cholesterol in HDLs may be esterified by the enzymatic activity of LCAT. The LCAT reaction occurs in two steps. After binding to a lipoprotein, LCAT cleaves the fatty acid in the sn-2 position of phosphatidylcholine and transfers it onto a serine residue. Next, the fatty acid is transesterified to the 3-β-hydroxyl group on the A-ring of cholesterol to form cholesterol ester. Cholesteryl esters formed by LCAT partition, which are more hydrophobic than free cholesterol, are transferred from the surface of lipoproteins to the hydrophobic core. This process converts pre-β HDL to HDL_2_ and HDL_3_ particles, which are the major HDL species found in plasma and which represent larger, spherical-shaped α-migrating forms of HDL. LCAT is important in the process of RCT by generating a gradient of free cholesterol from cells to HDL [79]. This effect of LCAT prevents the back exchange of cholesterol by passive diffusion from HDL to peripheral cells and thus is believed to promote net removal of cholesterol from peripheral cells to HDL. Without ongoing esterification of cholesterol, the capacity of HDL to remove and bind additional cholesterol would eventually be diminished. Two lipases, endothelial lipase (EL) and HL, are the complete opposite of LCAT in HDL metabolism. HL and EL are members of the triglyceride lipase family, which also includes LPL [80,81]. EL has high phospholipase A1 activity and remodels HDL into small particles, whereas HL is more effective in hydrolyzing triglycerides [82]. Although HL causes a remodeling of HDL into smaller particles, it also promotes the release of lipid-poor apoA-I [83]. The combined functions of HL and EL have a significant effect on plasma HDL-C levels [84,85,86,87]. HL- and EL-deficient mice were studied to demonstrate that the magnitude of macrophage-derived [^3^H] cholesterol in feces was increased, which promoted macrophage-to-feces RCT, and its ability to protect against LDL oxidation was enhanced [88].

##### Part Three: The Exchange of CE (Cholesteryl Esters) Mediated by CETP

CETP is a hydrophobic glycoprotein that is synthesized in several tissues but mainly in the liver. It facilitates the exchange of cholesteryl esters and triglycerides between HDL and apoB-containing particles (LDL, IDL, VLDL) and represents a major branching point for RCT [7,8]. This results in the recycling of cholesterol and an increasing attenuation in blood circulation, with the potential to go back into the artery wall. Two major observations were made using many novel methods, such as innovative X-ray crystallographic, electron microscopic (EM), and bioinformatics observations: (i) CETP connects with or forms bridges between two lipoproteins, e.g., HDL and LDL, with resultant neutral lipid transfer; and (ii) CETP appears to contain a hydrophobic tunnel along its entire long axis capable of neutral lipid transfer. These observations may clearly explain the relationships between CETP interactions with lipoproteins and the lipid transfer processes [89]. Most CEs derived from LCAT do not return to the liver via the HDL SR-B1 pathway but, rather, through more atherogenic pathways. CETP mediates the transfer of most CE from HDL to VLDL or to other more atherogenic intermediate-density lipoproteins and remnants, and the transfer of triglycerides from VLDL-1 to HDL results in larger, relatively triglyceride-enriched LDL species [90]. Transfer of CE from HDL directly to LDL by CETP could also be antiatherogenic if the LDL is cleared by the liver LDL receptor. Another transfer protein in this part of RCT, PLTP, transfers phospholipids between VLDL and HDL [91]. PLTP is one of the main modulators of plasma HDL size, composition and function [92] and one of the major modulators of HDL metabolism in plasma [93]. The level of HDL or HDL production is dramatically decreased in PLTP*^–^*^/*–*^ mice [94,95], and PLTP deficiency attenuates plaque accumulation in different atherosclerotic models. This indicates that PLTP plays an important role in atherogenesis, and its function goes well beyond that of transferring phospholipids between lipoprotein particles [96,97].

##### Part Four: Catabolism of HDL Cholesterol in Biliary Pathway

After efflux, cholesterol in HDLs may be esterified by the enzymatic activity of LCAT whereupon HDLs can deliver the excess cholesterol from peripheral cells back to the liver in in distinct ways: HDL cholesteryl esters, but not the protein component of HDLs, are selectively taken up into the liver via SR-BI. Ultimately, cholesterol is excreted from the liver into the bile, either directly as free cholesterol or after conversion into bile acids, and eliminated from the body via the feces. In humans, HDL-C can be metabolized by the liver via another pathway: CETP exchanges of HDL CE for triglycerides in apoB-containing lipoproteins, followed by hepatic uptake mediated by LDL-r. An LDL-r deficiency in mice substantially decreases selective HDL CE uptake by liver and adrenals. Thus, LDL-r expression has a substantial impact on HDL metabolism in mice [98].

Via the classic RCT pathway, excessive cholesterol collected from peripheral tissues, which is delivered back to the liver, is followed by biliary secretion and elimination via the feces. In addition to the traditional RCT-mediated biliary pathway, in the last few years, direct trans-intestinal excretion of plasma-derived cholesterol (TICE) was shown to contribute substantially to fecal neutral sterol (FNS) excretion in mice, describing the transport of cholesterol from blood to the intestinal lumen directly via enterocytes. The TICE pathway was called a nonhepatobiliary-related route, which has been shown to have a high degree of correlation with the main contributors Niemann-Pick disease, type C1/2 (NPC1/2), ABCG5/G8, LDL-r, and LXR [99,100,101]. The application of PPAR δ agonist and LXR agonists, have been shown to stimulate the process of TICE [102]. In the RCT pathway, HDL plays an important role. In contrast, there is evidence from animal experiments that HDL plays an essential role in TICE [103].

#### 2.2.2. Antioxidant Properties of High-Density Lipoprotein

LDL is one of the main causes of AS. Oxidation of LDL yields a more pro-atherogenic particle, and numerous studies have found that HDLs are capable of impeding oxidative changes in LDL. HDL exhibits potent antioxidant activity, which may arise from synergy in the inactivation of oxidized LDL lipids by enzymatic and nonenzymatic mechanisms, in part reflecting distinct intrinsic physicochemical properties [104]. The anti-oxidative properties of HDL critically involve HDL-associated enzymes, such as paraoxonase 1 (PON1), lipoprotein-associated phospholipase A2 (Lp-PLA2), and LCAT, which have been reported to hydrolyze oxidized phospholipids into lyso-phosphatidylcholine [105,106,107,108,109]. In addition, HDL carries glutathione selenoperoxidase, which can reduce lipid peroxide (LOOH) to the corresponding hydroxides and thereby detoxify them [110]. ApoA-I can remove oxidized lipids from LDL, suggesting that HDL can function as an acceptor of oxidized lipids. In cell culture experiments, apoA-I removes lipids from LDL and thereby makes LDL resistant to vascular cell-mediated oxidation and prevents oxidized LDL-induced monocyte adherence and chemotaxis [108]. Other HDL apolipoproteins, such as apoA-II, apoA-IV, apoE, and apoJ, also function as antioxidants *in vitro*.

#### 2.2.3. HDL in the Endothelium

Traditionally, the endothelium has been considered to be an inert component of the vessel wall. Injury to the endothelium results in deleterious alterations of endothelial physiology, also referred to as endothelial dysfunction, which represents a key early step in the development of an atherosclerotic lesion and is implicated in the malignant development that follows. It has become evident that HDL from healthy subjects can exert direct potential atheroprotective effects on endothelial cells and can positively affect several endothelial functions in the regulation of vascular tone, inflammation [111] and endothelial oxidant stress [112], which is associated with activation of NO synthesis by HDL. NO is an endothelium-derived signaling molecule that activates guanylate cyclase in vascular smooth muscle cells (SMCs) to induce relaxation, which is generated by a constitutive eNOS. Notably, HDLs also beneficially affect the vasculature by promoting endothelial cell survival. In endothelial cells, HDLs still maintain anti-thrombotic functions, mainly inhibiting thrombin-induced tissue factor, mediating the extrinsic coagulation pathway, and stimulating the activation of the anticoagulant proteins C and S.

#### 2.2.4. Anti-inflammatory Properties of HDL

HDL and apoA I are believed to protect against the development of AS, in part, which is closely related to their anti-inflammatory function. The anti-inflammatory properties of HDL encompass suppression of macrophage inflammatory cytokine production and inhibition of the expression of endothelial cell adhesion molecules that promote the entry of monocytes and neutrophils into arteries. HDLs potently block murine experimental endotoxinemia, indicating that HDLs bind lipopolysaccharides (LPS), which leads to lower systemic proinflammatory cytokine levels and improved survival rates [113]. Up-regulation of adhesion molecules (E-selectin, ICAM-1, and VCAM-1) and secretion of pro-inflammatory mediators (monocyte chemoattractant 1, MCP-1) lead to the activation of the vascular endothelium and induce AS.

## 3. Novel Therapies with HDL as the Key Components

### 3.1. Novel Pharmacotherapeutic Strategies in Increasing HDL

#### 3.1.1. Infusions of Special HDL

One approach to increase the serum levels of HDL is to infuse reconstituted HDL (rHDL) or recombinant HDL particles into the circulation rather than by increasing HDL indirectly by modulating HDL metabolism. CSL-111 is a reconstituted HDL-particle comprising both human apoA-1 and soybean phosphatidylcholine. CSL-112 is being evaluated in phase II trials to improve the weakness of CSL-111. There is a benefit of CSL-111/CSL-112 infusions to increase HDL-C levels and up-regulation of cellular cholesterol efflux in patients with acute coronary syndrome (ACS). Some trials have demonstrated that infusions of recombinant HDL-containing apoA-I Milano and CER-001 significantly reduce or stabilize mean plaque atheroma volume and still exert greater anti-inflammatory effects in LDL-r*^–^*^/*–*^ mice [114,115,116]. However, most clinical studies have demonstrated that rapid clearance of CER-001 leads to a requirement for repeated administration due to the inability to achieve effective plasma concentrations [117]. Pegylation of apoA-I in rHDL markedly increases its plasma half-life and enhances its antiatherogenic properties *in vivo* [118].

#### 3.1.2. Autologous Delipidated HDL

Another novel approach to HDL therapeutics is to raise the levels of HDL particles by intravenous infusion with the use of autologous delipidated HDL. Preclinical evaluation of selective delipidated HDL in a nonhuman primate model of dyslipidemia achieved a significant 6.9% reduction in aortic atheroma volume, as assessed by intravenous ultrasound (IVUS) [119]. In a small clinical study in which 28 patients with ACS received five weekly infusions of delipidated HDL, the decreased total atheroma volume by 5.2% from baseline may be associated with selectively increased preβ-HDL [120].

### 3.2. Novel Pharmacotherapeutic Strategies in Four Steps of RCT

#### 3.2.1. First Part of RCT

##### ApoA-I Mimetic Peptides

ApoA-I mimetics, which are short synthetic peptides, mimic the amphipathic a-helix of apoA-I. Because the first apoA-I mimetic consisted of 18 amino acids, additional improved peptides were generated by increasing the number of phenylalanine residues on the hydrophobic face (referred to as 2F, 3F, 4F, 5F, 6F, and 7F) of the polypeptide. Two different types of apoA-I mimetic peptide 4F were used in clinical trials, including l-4F (the 4F peptide synthesized from l-amino acids) and d-4F (the 4F peptide synthesized from all d-amino acids). Compared with high plasma l-4F levels not improving HDL anti-inflammatory function, a single dose of d-4F was found to have a significant effect on the inflammatory index of HDL with modest oral bioavailability [10]. ABCA1-independent lipid efflux played a major role in this aspect of RCT. Recently, 5A, an asymmetric bihelical peptide based on 2F, had increased ABCA1-dependent cholesterol efflux and decreased hemolysis compared with its parent compound [121]. Furthermore, ATI-5261 synthetic peptide, like apoA-I mimetic peptides, successfully enhances cholesterol efflux from macrophages and reduces aortic AS, which exerts its effects similar to that of the role of HDL in reverse cholesterol transport in mice. Reservelogix-208 (RVX-208), an apoA-I upregulator that increases endogenous synthesis of apoA-I, has a unique mechanism of action related to epigenetically influencing the accessibility of apoA-I gene by the transcription machinery. Encouragingly, there is a progressive discovery that nanolipid particles containing multivalent peptides (HDL-like nanoparticles) promoted efficient cellular cholesterol efflux and were functionally superior to those derived from monomeric apoA-I mimetic peptides [122].

##### Regulators of ABCA1 and ABCG1

LXR and miR-33 (similars: miR-758, miR-26 and miR-106b) have been demonstrated to regulate macrophages ABCA1 and ABCG1. The activation of the former can upregulate the expression of two genes, which promote macrophage cholesterol efflux and augment intestinal HDL generation, while the opposite is true of the latter. In the animal experiments, LXR agonists exhibit a significant decrease of atherosclerotic plaque formation and exert anti-inflammatory characteristics [123]. The negative effects caused by hepatic LXR agonists, such as a fatty liver, will be overcome by more intestine-specific LXRα agonists that are still under preclinical investigation [124]. As an alternative to LXR agonism, gene- silencing approaches involving microRNA 33 are being explored to control gene expression at the post-transcriptional level [125]. An antisense oligonucleotide has been developed that effectively silences miR-33 (miRNA-33a/b) and increases ABCA1 and ABCG1 expression in macrophages. Studies in nonhuman primates showed that antisense oligonucleotides to miRNA-33a/b that induce ABCA1 expression as well as the expression of other proteins involved in fatty acid metabolism result in a 50% increase in HDL-C and a decrease in VLDL [126]. Recent studies in PPARγ-activated macrophages demonstrated that treatment with miR-613 leads to inhibition of cholesterol efflux by downregulating LXRα and ABCA1 [127]. This study sheds new insights into the possibility that the miR-613 inhibitor may serve as a novel therapy for the treatment of cholesterol metabolism diseases. Similarly, PPAR agonists also increase ABCA1 gene expression and target the LXR gene. PPARα (K-877), PPARγ (INT131) and PPARα/δ (GFT505) modulators play pivotal roles in ABCA1 gene expression and apoA-I secretion, and increase HDL-C in patients with hyperlipidemia [126,127]. Growing evidence suggests that MBX-8025, a promising PPARδ agonist targeting the ABCA1 gene and LXRα gene, with a greater binding potency, is under development. Additional dual PPARα/γ and PPARβ/δ agonists that are under development may play an important role in RCT pathway [78].

#### 3.2.2. Second Part of RCT

##### LCAT Agonists

LCAT plays a major role in this part of RCT, and mediates the esterification of free cholesterol located at the surface of lipoprotein particles. Several drug development approaches have recently been initiated for modulating LCAT activity. Recombinant human LCAT (rhLCAT) efficiently promotes the process of RCT, which induces a marked increase in HDL-C levels and the maturation of small preb-HDL into α-migrating particles in LCAT-deficient plasma [128]. Currently, recombinant LCAT (ACP-501) in Phase 1 clinical trials was shown to rapidly and substantially elevate the formation of cholesteryl esters, representing the promotion of RCT. It is reported that Apo AI-derived peptides present the ability to modulate LCAT activity, with a significant increase in 3H-cholesteryl ester production in a higher proportion at 0.3 mg/mL [129]. An early apoA-I mimetic peptide (ETC-642) promotes LCAT activation and has entered clinical development [130].

#### 3.2.3. Third Part of RCT

##### CETP Inhibitor

**Anacetrapib.** The Determining Efficacy and Tolerability of Anacetrapib has shown a 138% increase in HDL-C, a 40% reduction in LDL-C, and a 36% decrease in Lp(a) of patients with CAD. This results from the ability of anacetrapib to inhibit both heterotypic and homotypic CE transfer. A large phase III study, Randomized EValuationof the Effects of Anacetrapib Through Lipid-modification (REVEAL), will be completed in January 2017 [131].

**Evacetrapib.** Evacetrapib has a similar structure and mechanism of action as torcetrapib but, based on IC_50_ values, appears to be a more potent CETP inhibitor than torcetrapib or anacetrapib [132]. It is the most recently developed CETP-inhibitor currently undergoing a phase III CVD outcome trial. In a phase II study, high doses of evacetrapib do not elevate blood pressure in rats and do not induce aldosterone or cortisol biosynthesis in a human adrenal cortical carcinoma cell line [133]. The Assessment of Clinical Effects of Cholesteryl Ester Transfer Protein Inhibition With Evacetrapib in Patients at a High-Risk for Vascular Outcomes (ACCELERATE) phase III CVD outcome trial is recruiting.

**BAY 60-5521/TA-8995.** BAY 60-5521 and TA-8995 are two potential inhibitors of CETP. The clinical results suggest that these two agents are safe and well tolerated, with effective inhibition of CETP activity and increased high HDL-C [134,135].

**CETP ASO.** Antisense oligonucleotide inhibitor of CETP (CETP ASO) is associated with reductions in CETP mRNA and also shows an enhanced effect on macrophage RCT. It is reported that the CETP ASO does not act through binding and inactivating CETP associated with the HDL particle. Instead, it specifically targets and degrades CETP mRNA. In hyperlipidemic, CETP transgenic, LDL-r*^–^*^/*–*^ mice, CETP ASO provided comparable reductions in HDL cholesterol, decreases in CETP activity with an enhanced effect on macrophage RCT, and results in less accumulation of aortic cholesterol [136]. CETP ASO could produce a unique therapeutic profile, distinct from the current CETP drugs being evaluated in late-stage clinical trials.

#### 3.2.4. Fourth Part of RCT

##### SR-BI Activators

SR-BI, the HDL receptor, is expressed on hepatocytes and facilitates selective absorption of cholesterol ester from HDL. The discovery and development of traditional drugs that can modulate SR-BI expression and/or activity are being investigated through high-throughput screening of chemical compound libraries. Endogenous or exogenous agents (such as natural products) and the detailed high-resolution molecular structure of SR-BI protein, the regulators of SR-BI activity, should provide new insights for the progress of non-genetic therapies to modulate HDL metabolism *in vivo*.

As an alternative to traditional drugs, gene therapeutics are being extensively explored. In general, miRNA-125a and miRNA-455 can bind to specific sites in the 3′ UTR of SR-BI mRNA and act as endogenous attenuators of SR-BI protein expression [137]. It is clear that activation of miRNA-125a and miRNA-455 shows a negative relationship with SR BI-mediated selective HDL uptake and SR-BI-supported steroid hormone synthesis. Therefore, inhibition of miRNA-125a and miRNA-455 may enhance SR-BI expression to alter the course of the atherogenic sequence by increasing plasma HDL-C flux. Furthermore, in a recent report, the level of SR-BI expression was repressed by miR-185, miR-96, and miR-223, individually, in HepG2 cells [138]. Inhibitions of these miRNAs were associated with increased SR-BI expression and reduced AS by facilitating RCT and cholesterol removal from the body.

### 3.3. Natural Drugs Associated with HDL at the Future Stage

Nature is an irrefutable source of inspiration for the modern man in many aspects. The observation and understanding of nature have allowed the development of new materials, new sources of energies, new drugs, *etc.* Specifically, natural products provide a great contribution to the development of new agents for the treatment of hyperlipidemic diseases. Compared with chemical drugs or other synthetically created products, natural products could more closely mimic physiological processes and may be more effective in treatment of cardiovascular diseases. There are many natural drugs for the treatment of AS associated with HDL, such as polysaccharide, sesamin, anthocyanins, 24(*S*)-Saringosterol and many others. Chitosan and its related products, which are polysaccharides, are used to enhance natural products and may represent promising strategies in the near future.

#### 3.3.1. Chitosan

Chitosan (CTS), the only positive alkaline polysaccharide extracted from shrimp or crab shells and exhibiting biocompatibility and a nontoxic nature, has the potential for hypolipidemic and weight loss activities. In 1980, the first article demonstrating that proper supplementation of chitosan to the diet seemed to be effective in lowering plasma cholesterol in rats was published [139]. And the meta-analysis by Baker *et al.*, based on six randomized placebo-controlled clinical trials in hypercholesterolemic patients has showed that chitosan only induced a decrease in total cholesterol level but without any significant changes in LDL-cholesterol, HDL-cholesterol or triglyceride concentrations [140]. Since then, the research of CTS in treatment of hyperlipemia has been extensively undertaken. It is reported that because CTS is a biodegradable carbohydrate polymer, it has been prepared in a variety of dosages for use in delivery systems in previous studies [141,142,143], and these new preparations were found to have a more effective capability of improving hyperlipidemia in rats [144,145].

#### 3.3.2. Chitosan Oligosaccharides

Chitosan oligosaccharides (COS) are depolymerized products of chitosan by either chemical or enzymatic hydrolysis. COS is a polymer of glucosamine with a number of bioactive properties. Recent studies have suggested that COS inhibits adipogenesis and promotes RCT through altered expression of a number of key regulators of lipid metabolism, including leptin [146], SR-BI and CYP7A1. It is reported that COS plays a positive role in the RCT pathway *in vivo* and stimulates hepatic LDL-r expression, inducing the LDL cholesterol lowering. Furthermore, chitosan oligosaccharides have anti-inflammatory functions similar to that of HDL, which downregulates the expression of E-selectin, ICAM-1 and IL-8 induced by LPS in endothelial cells through blockade of p38 MAPK and PI3K/Akt signaling pathways [147,148].

## 4. Discussion

HDL particles have various effects *in vitro* and *in vivo* that may protect arteries from chemical or biological hazards or facilitate repair of injuries. Nevertheless, HDL has not yet been successfully exploited for therapy. One possible reason may be the complexity of HDL particles, resulting in the physiological heterogeneity that contributes to the antiatherogenic functions of HDL. Moreover, for more than 50 years, HDL-C has been known as an independent clinical biomarker of cardiovascular risk. However, the failures of drugs to increase HDL-C drive scientists to search for new biomarkers to replace it, such as HDL functions, HDL size, and HDL-P. Among these, HDL functions will be a reliable diagnostic biomarker for the identification, personalized treatment stratification, and monitoring of patients at increased cardiovascular risk. The functionality of HDLs has two important roles. First, it promotes reverse cholesterol transport; second, it modulates inflammation and oxidation. HDL-based interventions in promoting the RCT process may be more promising. Meanwhile, some natural products with functions similar to that of HDL also play unique roles in the RCT process and will enter a new era in treatment of CVD in the near future (Table 2).

**Table 2:** Overview of Classes of HDL-based therapies. HDL-based therapies are an innovative approach against atherosclerosis. In this review, they can be summarized as the following three categories: strategies increasing HDL, strategies of RCT in four steps and natural drugs. ●: primary; ♦: secondary.

Strategies Increasing HDL		Strategies of RCT in Four Steps				Natural Drugs	
● Infusions of special HDL	♦ rHDL: CSL-111 CER-001 CSL-112	● **First part of RCT**		● **Second part of RCT**		● Polysaccharide:	Chitosan; Chitosan oligosaccharides
		ApoA-I mimetic peptides; ApoA-I upregulator		LCAT Agonists; ♦ rLCAT: ACP-501			
		LXR agonists; Mir-33; PPAR modulators				● Anthocyanins	
● Autologous delipidated HDL		● **Third part of RCT**		● **Fourth part of RCT**			
		♦ CETP inhibitor:	Anacetrapib; Evacetrapib; BAY 60-5521; TA-8995; CETP ASO	♦ SR-BI activators:	Traditional drugs; Gene therapeutics	● Sesamin	
						● 24(*S*)-Saringosterol	
						● Others	

## References

[1] Roger V.L., Go A.S., Lloyd-Jones D.M., Benjamin E.J., Berry J.D., Borden W.B., Bravata D.M., Dai S., Ford E.S., Fox C.S. (2012). Executive summary: Heart disease and stroke statistics-2012 update: A report from the American heart association. Circulation.

[2] Gordon T., Castelli W.P., Hjortland M.C., Kannel W.B., Dawber T.R. (1977). High density lipoprotein as a protective factor against coronary heart disease. The framingham study. Am. J. Med..

[3] Baigent C., Blackwell L., Emberson J., Holland L.E., Reith C., Bhala N., Peto R., Barnes E.H., Keech A., Simes J. (2010). Efficacy and safety of more intensive lowering of LDL cholesterol: A meta-analysis of data from 170,000 participants in 26 randomised trials. Lancet.

[4] Barter P., Gotto A.M., LaRosa J.C., Maroni J., Szarek M., Grundy S.M., Kastelein J.J., Bittner V., Fruchart J.C. (2007). HDL cholesterol, very low levels of LDL cholesterol, and cardiovascular events. N. Engl. J. Med..

[5] Dumitrescu L., Goodloe R., Bradford Y., Farber-Eger E., Boston J., Crawford D.C. (2015). The effects of electronic medical record phenotyping details on genetic association studies: HDL-C as a case study. Biodata Min..

[6] Remaley A.T., Norata G.D., Catapano A.L. (2014). Novel concepts in HDL pharmacology. Cardiovasc. Res..

[7] Schwartz G.G., Olsson A.G., Abt M., Ballantyne C.M., Barter P.J., Brumm J., Chaitman B.R., Holme I.M., Kallend D., Leiter L.A. (2012). Effects of dalcetrapib in patients with a recent acute coronary syndrome. N. Engl. J. Med..

[8] Barter P.J., Caulfield M., Eriksson M., Grundy S.M., Kastelein J.J., Komajda M., Lopez-Sendon J., Mosca L., Tardif J.C., Waters D.D. (2007). Effects of torcetrapib in patients at high risk for coronary events. N. Engl. J. Med..

[9] Marsche G., Saemann M.D., Heinemann A., Holzer M. (2013). Inflammation alters HDL composition and function: Implications for HDL-raising therapies. Pharmacol. Ther..

[10] Shah P.K. (2011). Atherosclerosis: Targeting endogenous apo AI—A new approach for raising HDL. Nat. Rev. Cardiol..

[11] Holleboom A.G., Jakulj L., Franssen R., Decaris J., Vergeer M., Koetsveld J., Luchoomun J., Glass A., Hellerstein M.K., Kastelein J.J. (2013). *In vivo* tissue cholesterol efflux is reduced in carriers of a mutation in APOA1. J. Lipid Res..

[12] Umemoto T., Han C.Y., Mitra P., Averill M.M., Tang C., Goodspeed L., Omer M., Subramanian S., Wang S., den Hartigh L.J. (2013). Apolipoprotein AI and high-density lipoprotein have anti-inflammatory effects on adipocytes via cholesterol transporters: ATP-binding cassette A-1, ATP-binding cassette G-1, and scavenger receptor B-1. Circ. Res..

[13] Navab M., Reddy S.T., van Lenten B.J., Fogelman A.M. (2011). HDL and cardiovascular disease: Atherogenic and atheroprotective mechanisms. Nat. Rev. Cardiol..

[14] Hewing B., Parathath S., Barrett T., Chung W.K., Astudillo Y.M., Hamada T., Ramkhelawon B., Tallant T.C., Yusufishaq M.S., Didonato J.A. (2014). Effects of native and myeloperoxidase-modified apolipoprotein A-I on reverse cholesterol transport and atherosclerosis in mice. Arterioscler. Thromb. Vasc. Biol..

[15] Rader D.J., Alexander E.T., Weibel G.L., Billheimer J., Rothblat G.H. (2009). The role of reverse cholesterol transport in animals and humans and relationship to atherosclerosis. J. Lipid Res..

[16] Hartman J., Frishman W.H. (2014). Inflammation and atherosclerosis: A review of the role of interleukin-6 in the development of atherosclerosis and the potential for targeted drug therapy. Cardiol. Rev..

[17] Glass C.K., Witztum J.L. (2001). Atherosclerosis. The road ahead. Cell.

[18] Libby P. (2002). Inflammation in atherosclerosis. Nature.

[19] Prasad V., Bonow R.O. (2011). The cardiovascular biomarker conundrum: Challenges and solutions. JAMA.

[20] The AIM-HIGH Investigators (2011). The role of niacin in raising high-density lipoprotein cholesterol to reduce cardiovascular events in patients with atherosclerotic cardiovascular disease and optimally treated low-density lipoprotein cholesterol: baseline characteristics of study participants. The Atherothrombosis Intervention in Metabolic syndrome with low HDL/high triglycerides: Impact on Global Health outcomes (AIM-HIGH) trial. Am. Heart J..

[21] Boden W.E., Probstfield J.L., Anderson T., Chaitman B.R., Desvignes-Nickens P., Koprowicz K., McBride R., Teo K., Weintraub W. (2011). Niacin in patients with low HDL cholesterol levels receiving intensive statin therapy. N. Engl. J. Med..

[22] Li C., Zhang W., Zhou F., Chen C., Zhou L., Li Y., Liu L., Pei F., Luo H., Hu Z. (2013). Cholesteryl ester transfer protein inhibitors in the treatment of dyslipidemia: A systematic review and meta-analysis. PLoS ONE.

[23] Boekholdt S.M., Arsenault B.J., Hovingh G.K., Mora S., Pedersen T.R., Larosa J.C., Welch K.M., Amarenco P., Demicco D.A., Tonkin A.M. (2013). Levels and changes of HDL cholesterol and apolipoprotein A-I in relation to risk of cardiovascular events among statin-treated patients: A meta-analysis. Circulation.

[24] Keene D., Price C., Shun-Shin M.J., Francis D.P. (2014). Effect on cardiovascular risk of high density lipoprotein targeted drug treatments niacin, fibrates, and CETP inhibitors: Meta-analysis of randomised controlled trials including 117,411 patients. BMJ.

[25] Voight B.F., Peloso G.M., Orho-Melander M., Frikke-Schmidt R., Barbalic M., Jensen M.K., Hindy G., Holm H., Ding E.L., Johnson T. (2012). Plasma HDL cholesterol and risk of myocardial infarction: a mendelian randomisation study. Lancet.

[26] Saely C.H., Vonbank A., Drexel H. (2010). HDL cholesterol and residual risk of first cardiovascular events. Lancet.

[27] Mora S., Glynn R.J., Boekholdt S.M., Nordestgaard B.G., Kastelein J.J., Ridker P.M. (2012). On-treatment non-high-density lipoprotein cholesterol, apolipoprotein B, triglycerides, and lipid ratios in relation to residual vascular risk after treatment with potent statin therapy: JUPITER (justification for the use of statins in prevention: An intervention trial evaluating rosuvastatin). J. Am. Coll. Cardiol..

[28] Rye K.A., Bursill C.A., Lambert G., Tabet F., Barter P.J. (2009). The metabolism and anti-atherogenic properties of HDL. J. Lipid Res..

[29] Barter P., Kastelein J., Nunn A., Hobbs R. (2003). High density lipoproteins (HDLs) and atherosclerosis: The unanswered questions. Atherosclerosis.

[30] Arsenault B.J., Lemieux I., Despres J.P., Gagnon P., Wareham N.J., Stroes E.S., Kastelein J.J., Khaw K.T., Boekholdt S.M. (2009). HDL particle size and the risk of coronary heart disease in apparently healthy men and women: the EPIC-Norfolk prospective population study. Atherosclerosis.

[31] Azevedo C.H., Wajngarten M., Prete A.C., Diament J., Maranhao R.C. (2011). Simultaneous transfer of cholesterol, triglycerides, and phospholipids to high-density lipoprotein in aging subjects with or without coronary artery disease. Clinics.

[32] Pascot A., Lemieux I., Prud'Homme D., Tremblay A., Nadeau A., Couillard C., Bergeron J., Lamarche B., Despres J.P. (2001). Reduced HDL particle size as an additional feature of the atherogenic dyslipidemia of abdominal obesity. J. Lipid Res..

[33] Du X.M., Kim M.J., Hou L., le Goff W., Chapman M.J., van Eck M., Curtiss L.K., Burnett J.R., Cartland S.P., Quinn C.M. (2015). HDL particle size is a critical determinant of ABCA1-mediated macrophage cellular cholesterol export. Circ. Res..

[34] Kim D.S., Burt A.A., Rosenthal E.A., Ranchalis J.E., Eintracht J.F., Hatsukami T.S., Furlong C.E., Marcovina S., Albers J.J., Jarvik G.P. (2014). HDL-3 is a superior predictor of carotid artery disease in a case-control cohort of 1725 participants. J. Am. Heart Assoc..

[35] Ridker P.M., Genest J., Boekholdt S.M., Libby P., Gotto A.M., Nordestgaard B.G., Mora S., MacFadyen J.G., Glynn R.J., Kastelein J.J. (2010). HDL cholesterol and residual risk of first cardiovascular events after treatment with potent statin therapy: An analysis from the JUPITER trial. Lancet.

[36] Mora S., Glynn R.J., Ridker P.M. (2013). High-density lipoprotein cholesterol, size, particle number, and residual vascular risk after potent statin therapy. Circulation.

[37] El H.K., Arsenault B.J., Franssen R., Despres J.P., Hovingh G.K., Stroes E.S., Otvos J.D., Wareham N.J., Kastelein J.J., Khaw K.T. (2009). High-density lipoprotein particle size and concentration and coronary risk. Ann. Intern. Med..

[38] Mora S., Otvos J.D., Rifai N., Rosenson R.S., Buring J.E., Ridker P.M. (2009). Lipoprotein particle profiles by nuclear magnetic resonance compared with standard lipids and apolipoproteins in predicting incident cardiovascular disease in women. Circulation.

[39] Ala-Korpela M., Soininen P., Savolainen M.J. (2009). Letter by Ala-Korpela et al regarding article, “Lipoprotein particle profiles by nuclear magnetic resonance compared with standard lipids and apolipoproteins in predicting incident cardiovascular disease in women”. Circulation.

[40] Hutchins P.M., Ronsein G.E., Monette J.S., Pamir N., Wimberger J., He Y., Anantharamaiah G.M., Kim D.S., Ranchalis J.E., Jarvik G.P. (2014). Quantification of HDL particle concentration by calibrated ion mobility analysis. Clin. Chem..

[41] Dahlback B., Nielsen L.B. (2006). Apolipoprotein M—A novel player in high-density lipoprotein metabolism and atherosclerosis. Curr. Opin. Lipidol..

[42] Elsoe S., Christoffersen C., Luchoomun J., Turner S., Nielsen L.B. (2013). Apolipoprotein M promotes mobilization of cellular cholesterol *in vivo*. Biochim. Biophys. Acta.

[43] Wolfrum C., Poy M.N., Stoffel M. (2005). Apolipoprotein M is required for prebeta-HDL formation and cholesterol efflux to HDL and protects against atherosclerosis. Nat. Med..

[44] Su W., Jiao G., Yang C., Ye Y. (2009). Evaluation of apolipoprotein M as a biomarker of coronary artery disease. Clin. Biochem..

[45] Borup A., Christensen P.M., Nielsen L.B., Christoffersen C. (2015). Apolipoprotein M in lipid metabolism and cardiometabolic diseases. Curr. Opin. Lipidol..

[46] Brea D., Sobrino T., Blanco M., Fraga M., Agulla J., Rodriguez-Yanez M., Rodriguez-Gonzalez R., Perez D.L.O.N., Leira R., Forteza J. (2009). Usefulness of haptoglobin and serum amyloid A proteins as biomarkers for atherothrombotic ischemic stroke diagnosis confirmation. Atherosclerosis.

[47] King V.L., Thompson J., Tannock L.R. (2011). Serum amyloid A in atherosclerosis. Curr. Opin. Lipidol..

[48] Liuzzo G., Biasucci L.M., Gallimore J.R., Grillo R.L., Rebuzzi A.G., Pepys M.B., Maseri A. (1994). The prognostic value of C-reactive protein and serum amyloid a protein in severe unstable angina. N. Engl. J. Med..

[49] Delanghe J.R., Langlois M.R., de Bacquer D., Mak R., Capel P., van Renterghem L., de Backer G. (2002). Discriminative value of serum amyloid A and other acute-phase proteins for coronary heart disease. Atherosclerosis.

[50] Lepedda A.J., Nieddu G., Zinellu E., de Muro P., Piredda F., Guarino A., Spirito R., Carta F., Turrini F., Formato M. (2013). Proteomic analysis of plasma-purified VLDL, LDL, and HDL fractions from atherosclerotic patients undergoing carotid endarterectomy: Identification of serum amyloid A as a potential marker. Oxid. Med. Cell. Longev..

[51] McMahon M., Grossman J., FitzGerald J., Dahlin-Lee E., Wallace D.J., Thong B.Y., Badsha H., Kalunian K., Charles C., Navab M. (2006). Proinflammatory high-density lipoprotein as a biomarker for atherosclerosis in patients with systemic lupus erythematosus and rheumatoid arthritis. Arthritis Rheum..

[52] Fung E.T., Thulasiraman V., Weinberger S.R., Dalmasso E.A. (2001). Protein biochips for differential profiling. Curr. Opin. Biotechnol..

[53] Issaq H.J., Veenstra T.D., Conrads T.P., Felschow D. (2002). The SELDI-TOF MS approach to proteomics: Protein profiling and biomarker identification. Biochem. Biophys. Res. Commun..

[54] Watanabe J., Chou K.J., Liao J.C., Miao Y., Meng H.H., Ge H., Grijalva V., Hama S., Kozak K., Buga G. (2007). Differential association of hemoglobin with proinflammatory high density lipoproteins in atherogenic/hyperlipidemic mice. A novel biomarker of atherosclerosis. J. Biol. Chem..

[55] Frohlich J., Al-Sarraf A. (2011). Cholesterol efflux capacity and atherosclerosis. N. Engl. J. Med..

[56] Uto-Kondo H., Ayaori M., Ogura M., Nakaya K., Ito M., Suzuki A., Takiguchi S., Yakushiji E., Terao Y., Ozasa H. (2010). Coffee consumption enhances high-density lipoprotein-mediated cholesterol efflux in macrophages. Circ. Res..

[57] Oram J.F., Lawn R.M., Garvin M.R., Wade D.P. (2000). ABCA1 is the cAMP-inducible apolipoprotein receptor that mediates cholesterol secretion from macrophages. J. Biol. Chem..

[58] Santamarina-Fojo S., Peterson K., Knapper C., Qiu Y., Freeman L., Cheng J.F., Osorio J., Remaley A., Yang X.P., Haudenschild C. (2000). Complete genomic sequence of the human ABCA1 gene: analysis of the human and mouse ATP-binding cassette A promoter. Proc. Natl. Acad. Sci. USA.

[59] Wang N., Lan D., Chen W., Matsuura F., Tall A.R. (2004). ATP-binding cassette transporters G1 and G4 mediate cellular cholesterol efflux to high-density lipoproteins. Proc. Natl. Acad. Sci. USA.

[60] Nakamura K., Kennedy M.A., Baldan A., Bojanic D.D., Lyons K., Edwards P.A. (2004). Expression and regulation of multiple murine ATP-binding cassette transporter G1 mRNAs/isoforms that stimulate cellular cholesterol efflux to high density lipoprotein. J. Biol. Chem..

[61] Yancey P.G., Bortnick A.E., Kellner-Weibel G., de la Llera-Moya M., Phillips M.C., Rothblat G.H. (2003). Importance of different pathways of cellular cholesterol efflux. Arterioscler. Thromb. Vasc. Biol..

[62] Dikkers A., Freak D.B.J., Annema W., Groen A.K., Tietge U.J. (2013). Scavenger receptor BI and ABCG5/G8 differentially impact biliary sterol secretion and reverse cholesterol transport in mice. Hepatology.

[63] Von Eckardstein A., Nofer J.R., Assmann G. (2001). High density lipoproteins and arteriosclerosis. Role of cholesterol efflux and reverse cholesterol transport. Arterioscler. Thromb. Vasc. Biol..

[64] Rosenson R.S., Brewer H.J., Davidson W.S., Fayad Z.A., Fuster V., Goldstein J., Hellerstein M., Jiang X.C., Phillips M.C., Rader D.J. (2012). Cholesterol efflux and atheroprotection: Advancing the concept of reverse cholesterol transport. Circulation.

[65] Joy T., Hegele R.A. (2009). The end of the road for CETP inhibitors after torcetrapib?. Curr. Opin. Cardiol..

[66] Wang N., Tall A.R. (2003). Regulation and mechanisms of ATP-binding cassette transporter A1-mediated cellular cholesterol efflux. Arterioscler. Thromb. Vasc. Biol..

[67] Curtiss L.K., Valenta D.T., Hime N.J., Rye K.A. (2006). What is so special about apolipoprotein AI in reverse cholesterol transport?. Arterioscler. Thromb. Vasc. Biol..

[68] Kontush A., Chapman M.J. (2006). Functionally defective high-density lipoprotein: A new therapeutic target at the crossroads of dyslipidemia, inflammation, and atherosclerosis. Pharmacol. Rev..

[69] Freeman S.R., Jin X., Anzinger J.J., Xu Q., Purushothaman S., Fessler M.B., Addadi L., Kruth H.S. (2014). ABCG1-mediated generation of extracellular cholesterol microdomains. J. Lipid Res..

[70] Song G.J., Kim S.M., Park K.H., Kim J., Choi I., Cho K.H. (2015). SR-BI mediates high density lipoprotein (HDL)-induced anti-inflammatory effect in macrophages. Biochem. Biophys. Res. Commun..

[71] Wang X., Collins H.L., Ranalletta M., Fuki I.V., Billheimer J.T., Rothblat G.H., Tall A.R., Rader D.J. (2007). Macrophage ABCA1 and ABCG1, but not SR-BI, promote macrophage reverse cholesterol transport *in vivo*. J. Clin. Investig..

[72] Daniil G., Zannis V.I., Chroni A. (2013). Effect of apoA-I Mutations in the capacity of reconstituted HDL to promote ABCG1-mediated cholesterol efflux. PLoS ONE.

[73] Westerterp M., Murphy A.J., Wang M., Pagler T.A., Vengrenyuk Y., Kappus M.S., Gorman D.J., Nagareddy P.R., Zhu X., Abramowicz S. (2013). Deficiency of ATP-binding cassette transporters A1 and G1 in macrophages increases inflammation and accelerates atherosclerosis in mice. Circ. Res..

[74] Bultel S., Helin L., Clavey V., Chinetti-Gbaguidi G., Rigamonti E., Colin M., Fruchart J.C., Staels B., Lestavel S. (2008). Liver X receptor activation induces the uptake of cholesteryl esters from high density lipoproteins in primary human macrophages. Arterioscler. Thromb. Vasc. Biol..

[75] Hanf R., Millatt L.J., Cariou B., Noel B., Rigou G., Delataille P., Daix V., Hum D.W., Staels B. (2014). The dual peroxisome proliferator-activated receptor α/δ agonist GFT505 exerts anti-diabetic effects in db/db mice without peroxisome proliferator-activated receptor gamma-associated adverse cardiac effects. Diabetes Vasc. Dis. Res..

[76] Colin S., Briand O., Touche V., Wouters K., Baron M., Pattou F., Hanf R., Tailleux A., Chinetti G., Staels B. (2013). Activation of intestinal peroxisome proliferator-activated receptor-α increases high-density lipoprotein production. Eur. Heart J..

[77] Sahebkar A., Chew G.T., Watts G.F. (2014). New peroxisome proliferator-activated receptor agonists: Potential treatments for atherogenic dyslipidemia and non-alcoholic fatty liver disease. Expert Opin. Pharmacother..

[78] Rousset X., Shamburek R., Vaisman B., Amar M., Remaley A.T. (2011). Lecithin cholesterol acyltransferase: An anti- or pro-atherogenic factor?. Curr. Atheroscler. Rep..

[79] Soran H., Hama S., Yadav R., Durrington P.N. (2012). HDL functionality. Curr. Opin. Lipidol..

[80] Olivecrona G., Olivecrona T. (2010). Triglyceride lipases and atherosclerosis. Curr. Opin. Lipidol..

[81] Chatterjee C., Sparks D.L. (2011). Hepatic lipase, high density lipoproteins, and hypertriglyceridemia. Am. J. Pathol..

[82] Yasuda T., Ishida T., Rader D.J. (2010). Update on the role of endothelial lipase in high-density lipoprotein metabolism, reverse cholesterol transport, and atherosclerosis. Circ. J..

[83] Annema W., Tietge U.J. (2011). Role of hepatic lipase and endothelial lipase in high-density lipoprotein-mediated reverse cholesterol transport. Curr. Atheroscler. Rep..

[84] Ishida T., Choi S., Kundu R.K., Hirata K., Rubin E.M., Cooper A.D., Quertermous T. (2003). Endothelial lipase is a major determinant of HDL level. J. Clin. Investig..

[85] Ruel I.L., Couture P., Cohn J.S., Bensadoun A., Marcil M., Lamarche B. (2004). Evidence that hepatic lipase deficiency in humans is not associated with proatherogenic changes in HDL composition and metabolism. J. Lipid Res..

[86] Lambert G., Amar M.J., Martin P., Fruchart-Najib J., Foger B., Shamburek R.D., Brewer H.J., Santamarina-Fojo S. (2000). Hepatic lipase deficiency decreases the selective uptake of HDL-cholesteryl esters *in vivo*. J. Lipid Res..

[87] Jaye M., Lynch K.J., Krawiec J., Marchadier D., Maugeais C., Doan K., South V., Amin D., Perrone M., Rader D.J. (1999). A novel endothelial-derived lipase that modulates HDL metabolism. Nat. Genet..

[88] Escola-Gil J.C., Chen X., Julve J., Quesada H., Santos D., Metso J., Tous M., Jauhiainen M., Blanco-Vaca F. (2013). Hepatic lipase- and endothelial lipase-deficiency in mice promotes macrophage-to-feces RCT and HDL antioxidant properties. Biochim. Biophys. Acta.

[89] Zhang L., Yan F., Zhang S., Lei D., Charles M.A., Cavigiolio G., Oda M., Krauss R.M., Weisgraber K.H., Rye K.A. (2012). Structural basis of transfer between lipoproteins by cholesteryl ester transfer protein. Nat. Chem. Biol..

[90] Chapman M.J., le Goff W., Guerin M., Kontush A. (2010). Cholesteryl ester transfer protein: At the heart of the action of lipid-modulating therapy with statins, fibrates, niacin, and cholesteryl ester transfer protein inhibitors. Eur. Heart J..

[91] Rao R., Albers J.J., Wolfbauer G., Pownall H.J. (1997). Molecular and macromolecular specificity of human plasma phospholipid transfer protein. Biochemistry.

[92] Yu Y., Guo S., Feng Y., Feng L., Cui Y., Song G., Luo T., Zhang K., Wang Y., Jiang X.C. (2014). Phospholipid transfer protein deficiency decreases the content of S1P in HDL via the loss of its transfer capability. Lipids.

[93] Albers J.J., Vuletic S., Cheung M.C. (2012). Role of plasma phospholipid transfer protein in lipid and lipoprotein metabolism. Biochim. Biophys. Acta.

[94] Jiang X.C., Bruce C., Mar J., Lin M., Ji Y., Francone O.L., Tall A.R. (1999). Targeted mutation of plasma phospholipid transfer protein gene markedly reduces high-density lipoprotein levels. J. Clin. Investig..

[95] Yazdanyar A., Quan W., Jin W., Jiang X.C. (2013). Liver-specific phospholipid transfer protein deficiency reduces high-density lipoprotein and non-high-density lipoprotein production in mice. Arterioscler. Thromb. Vasc. Biol..

[96] Jiang X.C., Qin S., Qiao C., Kawano K., Lin M., Skold A., Xiao X., Tall A.R. (2001). Apolipoprotein B secretion and atherosclerosis are decreased in mice with phospholipid-transfer protein deficiency. Nat. Med..

[97] Luo Y., Shelly L., Sand T., Reidich B., Chang G., Macdougall M., Peakman M.C., Jiang X.C. (2010). Pharmacologic inhibition of phospholipid transfer protein activity reduces apolipoprotein-B secretion from hepatocytes. J. Pharmacol. Exp. Ther..

[98] Rinninger F., Heine M., Singaraja R., Hayden M., Brundert M., Ramakrishnan R., Heeren J. (2014). High density lipoprotein metabolism in low density lipoprotein receptor-deficient mice. J. Lipid Res..

[99] Van der Velde A.E., Vrins C.L., van den Oever K., Kunne C., Oude E.R., Kuipers F., Groen A.K. (2007). Direct intestinal cholesterol secretion contributes significantly to total fecal neutral sterol excretion in mice. Gastroenterology.

[100] Van der Velde A.E., Brufau G., Groen A.K. (2010). Transintestinal cholesterol efflux. Curr. Opin. Lipidol..

[101] Blanchard C., Moreau F., Cariou B., Le May C. (2014). Trans-intestinal cholesterol excretion (TICE): A new route for cholesterol excretion. Med. Sci..

[102] Vrins C.L., van der Velde A.E., van den Oever K., Levels J.H., Huet S., Oude E.R., Kuipers F., Groen A.K. (2009). Peroxisome proliferator-activated receptor delta activation leads to increased transintestinal cholesterol efflux. J. Lipid Res..

[103] Vrins C.L., Ottenhoff R., van den Oever K., de Waart D.R., Kruyt J.K., Zhao Y., van Berkel T.J., Havekes L.M., Aerts J.M., van Eck M. (2012). Trans-intestinal cholesterol efflux is not mediated through high density lipoprotein. J. Lipid Res..

[104] Kontush A., Chantepie S., Chapman M.J. (2003). Small, dense HDL particles exert potent protection of atherogenic LDL against oxidative stress. Arterioscler. Thromb. Vasc. Biol..

[105] Kontush A., Chapman M.J. (2010). Antiatherogenic function of HDL particle subpopulations: Focus on antioxidative activities. Curr. Opin. Lipidol..

[106] Mackness B., Mackness M. (2012). The antioxidant properties of high-density lipoproteins in atherosclerosis. Panminerva Med..

[107] Vohl M.C., Neville T.A., Kumarathasan R., Braschi S., Sparks D.L. (1999). A novel lecithin-cholesterol acyltransferase antioxidant activity prevents the formation of oxidized lipids during lipoprotein oxidation. Biochemistry.

[108] Navab M., Hama S.Y., Anantharamaiah G.M., Hassan K., Hough G.P., Watson A.D., Reddy S.T., Sevanian A., Fonarow G.C., Fogelman A.M. (2000). Normal high density lipoprotein inhibits three steps in the formation of mildly oxidized low density lipoprotein: Steps 2 and 3. J. Lipid Res..

[109] Turunen P., Jalkanen J., Heikura T., Puhakka H., Karppi J., Nyyssonen K., Yla-Herttuala S. (2004). Adenovirus-mediated gene transfer of Lp-PLA2 reduces LDL degradation and foam cell formation *in vitro*. J. Lipid Res..

[110] Chen N., Liu Y., Greiner C.D., Holtzman J.L. (2000). Physiologic concentrations of homocysteine inhibit the human plasma GSH peroxidase that reduces organic hydroperoxides. J. Lab. Clin. Med..

[111] Besler C., Heinrich K., Rohrer L., Doerries C., Riwanto M., Shih D.M., Chroni A., Yonekawa K., Stein S., Schaefer N. (2011). Mechanisms underlying adverse effects of HDL on eNOS-activating pathways in patients with coronary artery disease. J. Clin. Investig..

[112] Sorrentino S.A., Besler C., Rohrer L., Meyer M., Heinrich K., Bahlmann F.H., Mueller M., Horvath T., Doerries C., Heinemann M. (2010). Endothelial-vasoprotective effects of high-density lipoprotein are impaired in patients with type 2 diabetes mellitus but are improved after extended-release niacin therapy. Circulation.

[113] Levine D.M., Parker T.S., Donnelly T.M., Walsh A., Rubin A.L. (1993). *In vivo* protection against endotoxin by plasma high density lipoprotein. Proc. Natl. Acad. Sci. USA.

[114] Nissen S.E., Tsunoda T., Tuzcu E.M., Schoenhagen P., Cooper C.J., Yasin M., Eaton G.M., Lauer M.A., Sheldon W.S., Grines C.L. (2003). Effect of recombinant ApoA-I Milano on coronary atherosclerosis in patients with acute coronary syndromes: A randomized controlled trial. JAMA.

[115] Ibanez B., Giannarelli C., Cimmino G., Santos-Gallego C.G., Alique M., Pinero A., Vilahur G., Fuster V., Badimon L., Badimon J.J. (2012). Recombinant HDL(Milano) exerts greater anti-inflammatory and plaque stabilizing properties than HDL(wild-type). Atherosclerosis.

[116] Tardy C., Goffinet M., Boubekeur N., Ackermann R., Sy G., Bluteau A., Cholez G., Keyserling C., Lalwani N., Paolini J.F. (2014). CER-001, a HDL-mimetic, stimulates the reverse lipid transport and atherosclerosis regression in high cholesterol diet-fed LDL-receptor deficient mice. Atherosclerosis.

[117] Tardif J.C., Ballantyne C.M., Barter P., Dasseux J.L., Fayad Z.A., Guertin M.C., Kastelein J.J., Keyserling C., Klepp H., Koenig W. (2014). Effects of the high-density lipoprotein mimetic agent CER-001 on coronary atherosclerosis in patients with acute coronary syndromes: A randomized trial. Eur. Heart J..

[118] Murphy A.J., Funt S., Gorman D., Tall A.R., Wang N. (2013). Pegylation of high-density lipoprotein decreases plasma clearance and enhances antiatherogenic activity. Circ. Res..

[119] Waksman R., Torguson R., Kent K.M., Pichard A.D., Suddath W.O., Satler L.F., Martin B.D., Perlman T.J., Maltais J.A., Weissman N.J. (2010). A first-in-man, randomized, placebo-controlled study to evaluate the safety and feasibility of autologous delipidated high-density lipoprotein plasma infusions in patients with acute coronary syndrome. J. Am. Coll. Cardiol..

[120] Sacks F.M., Rudel L.L., Conner A., Akeefe H., Kostner G., Baki T., Rothblat G., de la Llera-Moya M., Asztalos B., Perlman T. (2009). Selective delipidation of plasma HDL enhances reverse cholesterol transport *in vivo*. J. Lipid Res..

[121] Van Capelleveen J.C., Brewer H.B., Kastelein J.J., Hovingh G.K. (2014). Novel therapies focused on the high-density lipoprotein particle. Circ. Res..

[122] Zhao Y., Imura T., Leman L.J., Curtiss L.K., Maryanoff B.E., Ghadiri M.R. (2013). Mimicry of high-density lipoprotein: Functional peptide-lipid nanoparticles based on multivalent peptide constructs. J. Am. Chem. Soc..

[123] Joseph S.B., Castrillo A., Laffitte B.A., Mangelsdorf D.J., Tontonoz P. (2003). Reciprocal regulation of inflammation and lipid metabolism by liver X receptors. Nat. Med..

[124] Lo S.G., Murzilli S., Salvatore L., D’Errico I., Petruzzelli M., Conca P., Jiang Z.Y., Calabresi L., Parini P., Moschetta A. (2010). Intestinal specific LXR activation stimulates reverse cholesterol transport and protects from atherosclerosis. Cell Metab..

[125] Bartel D.P. (2004). MicroRNAs: Genomics, biogenesis, mechanism, and function. Cell.

[126] Rayner K.J., Esau C.C., Hussain F.N., McDaniel A.L., Marshall S.M., van Gils J.M., Ray T.D., Sheedy F.J., Goedeke L., Liu X. (2011). Inhibition of miR-33a/b in non-human primates raises plasma HDL and lowers VLDL triglycerides. Nature.

[127] Zhao R., Feng J., He G. (2014). miR-613 regulates cholesterol efflux by targeting LXRα and ABCA1 in PPARβ activated THP-1 macrophages. Biochem. Biophys. Res. Commun..

[128] Simonelli S., Tinti C., Salvini L., Tinti L., Ossoli A., Vitali C., Sousa V., Orsini G., Nolli M.L., Franceschini G. (2013). Recombinant human LCAT normalizes plasma lipoprotein profile in LCAT deficiency. Biologicals.

[129] Aguilar-Espinosa S.L., Mendoza-Espinosa P., Delgado-Coello B., Mas-Oliva J. (2013). Lecithin cholesterol acyltransferase (LCAT) activity in the presence of Apo-AI-derived peptides exposed to disorder-order conformational transitions. Biochem. Biophys. Res. Commun..

[130] Barylski M., Toth P.P., Nikolic D., Banach M., Rizzo M., Montalto G. (2014). Emerging therapies for raising high-density lipoprotein cholesterol (HDL-C) and augmenting HDL particle functionality. Best Pract. Res. Clin. Endocrinol. Metab..

[131] Barter P.J., Rye K.A. (2012). Cholesteryl ester transfer protein inhibition as a strategy to reduce cardiovascular risk. J. Lipid Res..

[132] Mohammadpour A.H., Akhlaghi F. (2013). Future of cholesteryl ester transfer protein (CETP) inhibitors: A pharmacological perspective. Clin. Pharmacokinet..

[133] Cao G., Beyer T.P., Zhang Y., Schmidt R.J., Chen Y.Q., Cockerham S.L., Zimmerman K.M., Karathanasis S.K., Cannady E.A., Fields T. (2011). Evacetrapib is a novel, potent, and selective inhibitor of cholesteryl ester transfer protein that elevates HDL cholesterol without inducing aldosterone or increasing blood pressure. J. Lipid Res..

[134] Ford J., Lawson M., Fowler D., Maruyama N., Mito S., Tomiyasu K., Kinoshita S., Suzuki C., Kawaguchi A., Round P. (2014). Tolerability, pharmacokinetics and pharmacodynamics of TA-8995, a selective cholesteryl ester transfer protein (CETP) inhibitor, in healthy subjects. Br. J. Clin. Pharmacol..

[135] Boettcher M.F., Heinig R., Schmeck C., Kohlsdorfer C., Ludwig M., Schaefer A., Gelfert-Peukert S., Wensing G., Weber O. (2012). Single dose pharmacokinetics, pharmacodynamics, tolerability and safety of BAY 60–5521, a potent inhibitor of cholesteryl ester transfer protein. Br. J. Clin. Pharmacol..

[136] Bell T.R., Graham M.J., Lee R.G., Mullick A.E., Fu W., Norris D., Crooke R.M. (2013). Antisense oligonucleotide inhibition of cholesteryl ester transfer protein enhances RCT in hyperlipidemic, CETP transgenic, LDLr*^–^*^/*–*^ mice. J. Lipid Res..

[137] Hu Z., Shen W.J., Kraemer F.B., Azhar S. (2012). MicroRNAs 125a and 455 repress lipoprotein-supported steroidogenesis by targeting scavenger receptor class B type I in steroidogenic cells. Mol. Cell. Biol..

[138] Wang L., Jia X.J., Jiang H.J., Du Y., Yang F., Si S.Y., Hong B. (2013). MicroRNAs 185, 96, and 223 repress selective high-density lipoprotein cholesterol uptake through posttranscriptional inhibition. Mol. Cell. Biol..

[139] Sugano M., Fujikawa T., Hiratsuji Y., Nakashima K., Fukuda N., Hasegawa Y. (1980). A novel use of chitosan as a hypocholesterolemic agent in rats. Am. J. Clin. Nutr..

[140] Baker W.L., Tercius A., Anglade M., White C.M., Coleman C.I. (2009). A meta-analysis evaluating the impact of chitosan on serum lipids in hypercholesterolemic patients. Ann. Nutr. Metab..

[141] Su Z.Q., Wu S.H., Zhang H.L., Feng Y.F. (2010). Development and validation of an improved Bradford method for determination of insulin from chitosan nanoparticulate systems. Pharm. Biol..

[142] Tan S., Gao B., Tao Y., Guo J., Su Z.Q. (2014). Antiobese effects of capsaicin-chitosan microsphere (CCMS) in obese rats induced by high fat diet. J. Agric. Food Chem..

[143] Chen J., Huang G.D., Tan S.R., Guo J., Su Z.Q. (2013). The preparation of capsaicin-chitosan microspheres (CCMS) enteric coated tablets. Int. J. Mol. Sci..

[144] Tao Y., Zhang H.L., Hu Y.M., Wan S., Su Z.Q. (2013). Preparation of chitosan and water-soluble chitosan microspheres via spray-drying method to lower blood lipids in rats fed with high-fat diets. Int. J. Mol. Sci..

[145] Pan H., Guo J., Su Z. (2014). Advances in understanding the interrelations between leptin resistance and obesity. Physiol. Behav..

[146] Zhang H.L., Tao Y., Guo J., Hu Y.M., Su Z.Q. (2011). Hypolipidemic effects of chitosan nanoparticles in hyperlipidemia rats induced by high fat diet. Int. Immunopharmacol..

[147] Li Y., Xu Q., Wei P., Cheng L., Peng Q., Li S., Yin H., Du Y. (2014). Chitosan oligosaccharides downregulate the expression of E-selectin and ICAM-1 induced by LPS in endothelial cells by inhibiting MAP kinase signaling. Int. J. Mol. Med..

[148] Liu H.T., Huang P., Ma P., Liu Q.S., Yu C., Du Y.G. (2011). Chitosan oligosaccharides suppress LPS-induced IL-8 expression in human umbilical vein endothelial cells through blockade of p38 and Akt protein kinases. Acta Pharmacol. Sin..

